# PKCι and YAP1 are crucial in promoting pancreatic tumorigenesis

**DOI:** 10.18632/oncotarget.25127

**Published:** 2018-08-28

**Authors:** Peipei Wang, Dapeng Wei, Hongmei Zhang, Jiao Chen, Dingding Zhang, Suthakar Ganapathy, Pauline Isakson, Changyan Chen, Tongbo Zhu

**Affiliations:** ^1^ Department of Immunology, West China School of Basic Medical Sciences and Forensic Medicine, Sichuan University, Chengdu, Sichuan 610041, P. R. China; ^2^ State Key Laboratory of Oral Disease, National Clinical Research Center for Oral Diseases, West China Hospital of Sichuan University, Chengdu, Sichuan 610041, P. R. China; ^3^ Sichuan Provincial Key Laboratory for Disease Gene Study, Hospital of University of Electronic Science and Technology and Sichuan Provincial People’s Hospital, University of Electronic Science and Technology of China, Chengdu, Sichuan 610072, P. R. China; ^4^ The Center of Drug Discovery, Northeastern University, Boston, MA 02115, USA; ^5^ Clinical Immunology and Transfusion Medicine, Sahlgrenska University Hospital, Göteborg 41345, Sweden

**Keywords:** Kras, PKCι, YAP1, survival, PDAC

## Abstract

Pancreatic ductal adenocarcinoma (PDAC) is a fatal malignant disease with 5-year survival rate of less than 6%. Activating mutations of *Kras* (*mu-Kras*) are often detected in most of PDAC patients. Although it has been known that oncogenic *Kras* is the driver of pancreatic cancer initiation and development, the underlying mechanisms by which *mu-Kras* promotes PDAC remain poorly understood. Here, we identify that PKCι is one of the crucial factors for supporting the survival of pancreatic cancer cells expressing *mu-Kras*. Our study demonstrates that after the knockdown of PKCι, the expression of the transcriptional co-activator YAP1 is decreased, which hinders the expression of the downstream target gene *Mcl-1*, and subsequently sensitizes pancreatic cancer MiaPaCa and PANC-1 cells experssing *mu-Kras* to apoptosis. In comparison, the suppression of PKCι has little impact on the viability of non-neoplastic pancreatic HPDE6-C7 cells. Moreover, the transient overexpression of oncogenic Kras in HPDE6-C7 elevates the expression of PKCι and YAP1 concomitantly. The upregulated YAP1 in HPDE6-C7/ *mu-Kras* cells is abolished once PKCι is suppressed, suggesting the linear relationship among mu-Kras, PKCι and YAP1. This phenomenon is further proven by the co-upregulation of PKCι and YAP1 in HPDE6-C7 cells stably transfected with *mu-Kras*. Taken together, our findings suggest that PKCι acts through promoting YAP1 function to promote the survival of pancreatic cancer cells expressing *mu-Kras*. It appears that targeting PKCι-YAP1 signaling is a feasible strategy for developing new therapeutics for treating pancreatic cancer patients.

## INTRODUCTION

Despite extensive research efforts, pancreatic ductal adenocarcinoma (PDAC) remains to be one of the most incurable lethal cancers with less than 6% patients surviving more than 5 years [1], a survival rate that hardly improves over the past few decades. The poor prognosis is attributed to the lack of diagnostic markers, insensitivity to radiotherapy, as well as limited and unsustained responsiveness to the available targeted chemotherapies. Although mutant *Kras*, a member of the *Ras* gene family and the most prevalent genetic alteration in cancer, has been identified in >90% PDAC and documented to be indispensable in driving the initiation and development of PDAC [2, 3], and recent advances in the development of mu-Kras specific inhibitor have offered some hope in targeting Kras protein for treatment [48], to date no pharmacological approaches that suppress the activity of this mutant protein have reached the clinic, compelling further delving into the signaling pathways engaged by Kras to identify alternative targets that are essential for PDAC maintenance and amenable to therapeutic intervention.

Protein kinase C (PKC) is a family of serine/threonine kinases that are classified into conventional, novel and atypical PKC subgroups according to their structures and the co-factors they need for activation. A variety of the PKC family members, for instance the conventional PKC isozymes α and β, and the novel PKC isozyme δ, have been illustrated to function coordinately to support the survival of cells carrying hyperactive Ras [4]. The two atypical PKC isozymes, ι and ζ, have also been defined as pro-proliferative in a diversity of cancers, with PKCι being recognized as an oncogene that plays pivotal roles in the transformation of numerous tumors including PDAC [5–7]. Nonetheless, the interaction between PKCι and Kras, as well as its contribution to the PDAC tumorigenesis and maintenance is not fully elucidated.

As a kinase, PKCι is capable of phosphorylating various substrates to trigger or modulate a panoply of cellular signaling pathways. In the evergrowing list of PKCι’s downstream effectors, Yes-associated protein 1 (YAP1, also known as YAP or YAP65) has drawn tremendous attention recently for its vital roles in transducing signals from the Hippo pathway and its implication in oncogenesis. *YAP1* gene locus 11q22 is found amplified in myriad tumors spanning liver, lung, esophagus, mammary gland carcinomas, oral squamous cell carcinoma and medulloblastomas [8–14], whereas YAP1 overexpression has been identified in lung, colorectal, hepatocellular, ovarian, prostate and pancreatic cancer cells [15–17]. Increased YAP1 level enhances anchorage-independent cell growth, cell migration, epithelial-mesenchymal transition (EMT) and induces stem-cell properties in mammary or pancreatic epithelial cells [13, 15, 18–21], while disruption of YAP1 signaling reverts these characteristics, pointing to an oncogenic role of YAP1 in tumors, especially those of epithelial origin. The pro-proliferative feature of YAP1 can be explained by its function as a transcriptional co-activator. Through binding to different transcription factors, YAP1 initiates the transcription of a wide spectrum of target genes, of which lots are proliferation-related. When YAP1 is phosphorylated by large tumor suppressor (Lats), a member of Hippo pathway, it is sequestered in cytoplasm and marked for proteosome-mediated degradation, resulting in blockade of YAP1-mediated transcription.

The involvement of YAP1 in Kras-driven oncogenic signaling has been evident in multiple studies. Zhang *et al.* discovered that YAP1 was upregulated and essential for the proliferation and progression of PDAC carrying aberrant Kras [22]. The association of YAP1 with PKCι is also witnessed in ovarian cancer cells. Recently, Wang *et al.* found that PKCι phosphorylated angiomotin (AMOT) and dissociated AMOT from YAP1, leading to YAP1 nuclear translocation and activation in ovarian cancer cells [23]. However, the interplays among these three molecules, Kras, PKCι and YAP1 have not been investigated. Here in this study, we report a discovery that hyperactive Kras upregulates the expression of YAP1 through PKCι to support the development and maintenance of PDAC. Interference with this signaling pathway restricts the growth and elicits apoptosis in PDAC cells. This discovery provides molecular insight into the PKCι-mediated cell survival in PDAC cells carrying aberrant Kras.

## RESULTS

### Knocking down atypical protein kinase C isozyme PKCι inhibits the growth and induces apoptosis in PDAC cells harboring aberrant Kras

**Figure 1:**
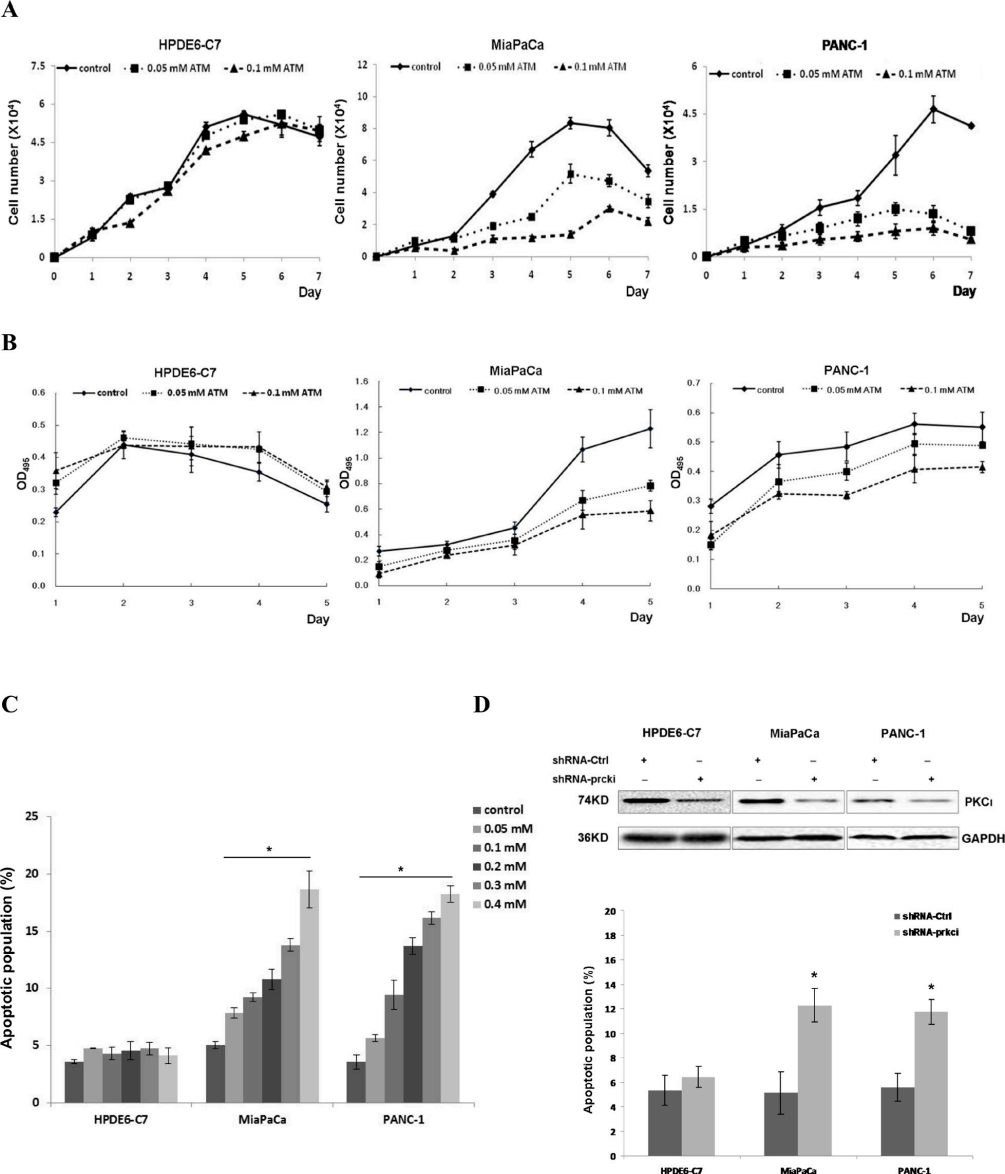
PKCι inhibition suppresses the growth and induces apoptosis of pancreatic cancer MiaPaCa and PANC-1 cells harboring aberrant Kras, while showing little effect on non-neoplastic pancreatic epithelial HPDE6-C7 cells containing normal Kras (**A**) Representative growth curves of HPDE6-C7, MiaPaCa and PANC-1 in presence of control diluent, 0.05 or 0.1 mM ATM. Cells were counted in triplicate wells at each time point and the result represents three independent experiments (*p* < 0.05). (**B)** Proliferation of HPDE6-C7, MiaPaCa or PANC-1 treated with control diluent, 0.05 or 0.1 mM ATM as determined by MTT colorimetric assay. Error bars represent SD of 3 replicates samples (*p* < 0.05). (**C**) Quantification of apoptotic cells induced by increasing concentrations of ATM in HPDE6-C7, MiaPaCa and PANC-1. The experiments were repeated at least five times (Error bars, ± SD. *n =* 5 per time point. ^*^p < 0.05). (**D**) Upper panels: Immunoblot analysis of PKCι expression in HPDE6-C7, MiaPaCa and PANC-1 infected with lentivirus producing non-targeting scrambled shRNA (shRNA-Ctrl) or shRNA targeting human PKCι (shRNA-prkci). Lower panel: Quantification of apoptotic cells in HPDE6-C7, MiaPaCa and PANC-1 expressing lentiviral shRNA-Ctrl or shRNA-prkci. Data represent mean of 5 replicates ± SD. ^*^p < 0.05.

In an effort to identify the PKC isozyme that may be the synthetic lethal partner of Kras in PDAC, we treated the MiaPaCa and PANC-1 cells with an assortment of pharmacological inhibitors against different PKC isozymes and found that aurothiomalate (ATM), a well-documented inhibitor to the atypical PKC isozyme ι, could suppress the growth and elicit apoptosis in these two cell lines in a dose-dependent manner (Figure 1A–1C). In contrast, when we treated the non-neoplastic pancreatic epithelium HPDE6-C7 cells carrying normal Kras with various concentrations of ATM ranging from 0.05 mM to 0.4 mM, we didn’t notice any growth inhibition or increased apoptotic population in ATM-treated HPDE6-C7 compared with their control diluent-treated counterparts (Figure 1A–1C). These results suggest that PKCι suppression by ATM inhibits the growth and elicits apoptosis in PDAC cells carrying mutant Kras.

Next, we knocked down the expression of PKCι in these three cell lines using a lentiviral short hairpin RNA (denoted as shRNA-prkci) and assessed the effects of genetic depletion of PKCι on the survival of these cells. The efficacy of shRNA-prkci was confirmed by immunoblot and qRT-PCR (Figures 1D, 3B–3D). In accordance with our initial findings, PKCι depletion led to increased apoptotic population in MiaPaCa and PANC-1, yet left HPDE6-C7 almost intact (Figure 1D). These results suggest that PKCι inhibition impairs the growth and results in apoptosis in PDAC cells harboring aberrant Kras, while having little effect on non-neoplastic pancreatic cells containing normal Kras.

### The expression of YAP1 is reduced upon PKCι inhibition in PDAC cells harboring aberrant Kras

**Figure 2:**
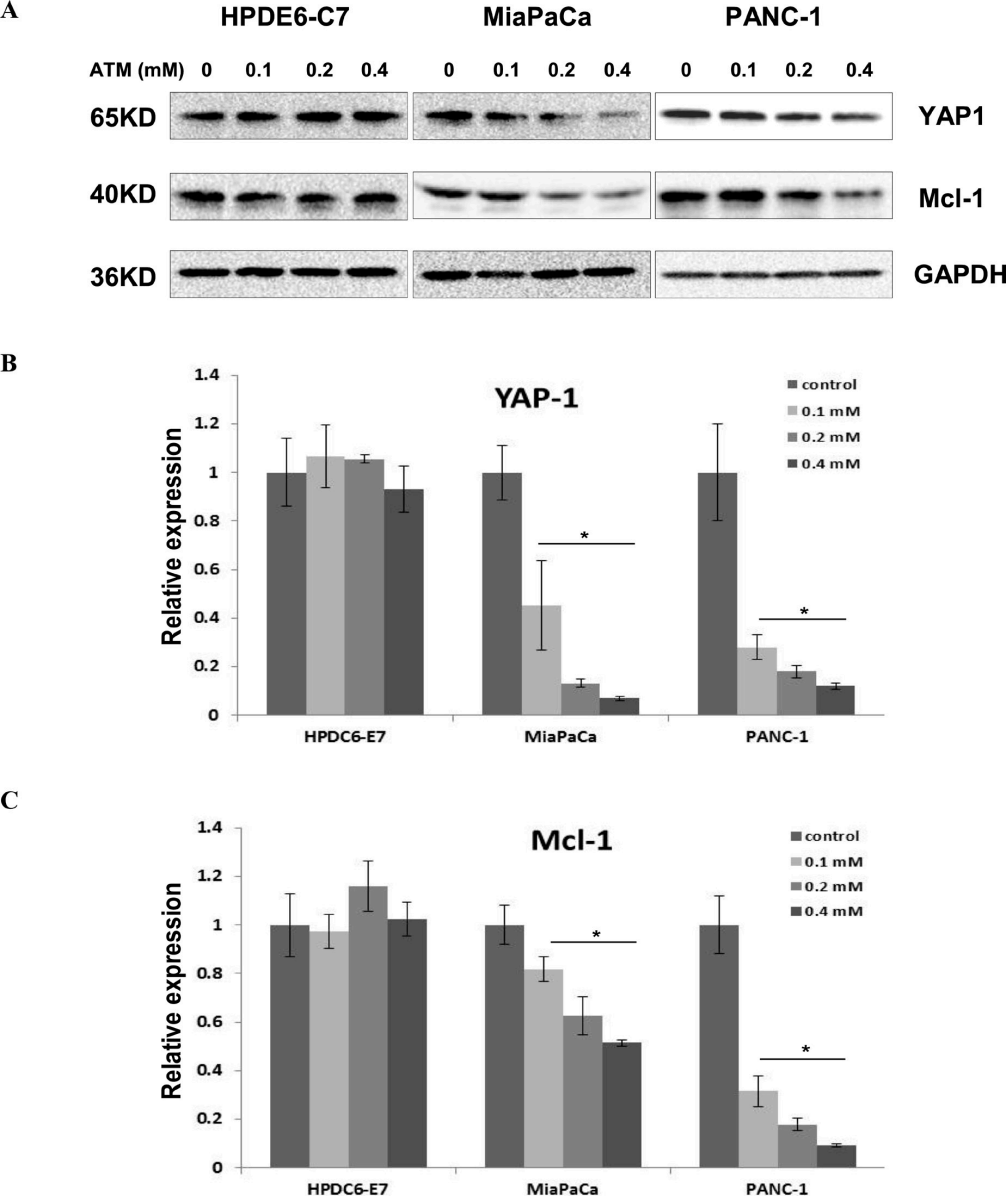
YAP1 and its downstream target Mcl-1 are downregulated in MiaPaCa and PANC-1 cells, but remain intact in HPDE6-C7 cells subjected to ATM treatment (**A**) Immunoblot analysis of YAP1 and Mcl-1 expression in HPDE6-C7, MiaPaCa and PANC-1 treated with increasing concentrations of ATM. (**B**) qRT-PCR analysis of YAP1 and (**C**) Mcl-1 levels in HPDE6-C7, MiaPaCa and PANC-1 in presence of increasing concentrations of ATM. Relative mRNA expression levels are normalized to reference gene and data are shown as fold change versus control (Error bars, ± SD. *n =* 3. ^*^p < 0.05).

To elucidate the mechanisms underlying the PKCι-mediated growth inhibition and cell death in PDAC cells, we examined the expression of a plethora of signaling molecules downstream of PKCι by immunoblot and qRT-PCR (data not shown). The results showed that YAP1, a transcriptional co-activator was downregulated in response to ATM treatment in a dose-dependent fashion in MiaPaCa and PANC-1 cells, while displaying little variation in ATM-treated HPDE6-C7 cells (Figure 2A and 2B), implying that YAP1 might be involved in the cellular effects exerted by PKCι. In addition, the level of Mcl-1, a protein related to cell proliferation and reported to be targeted by YAP1, declined along with YAP1 in MiaPaCa and PANC-1, but remained constant in HPDE6-C7 cells exposed to ATM (Figure 2A and 2C), indicating Mcl-1 is a potential effector downstream of PKCι-YAP1 signaling in PDAC cells with aberrant Kras.

**Figure 3:**
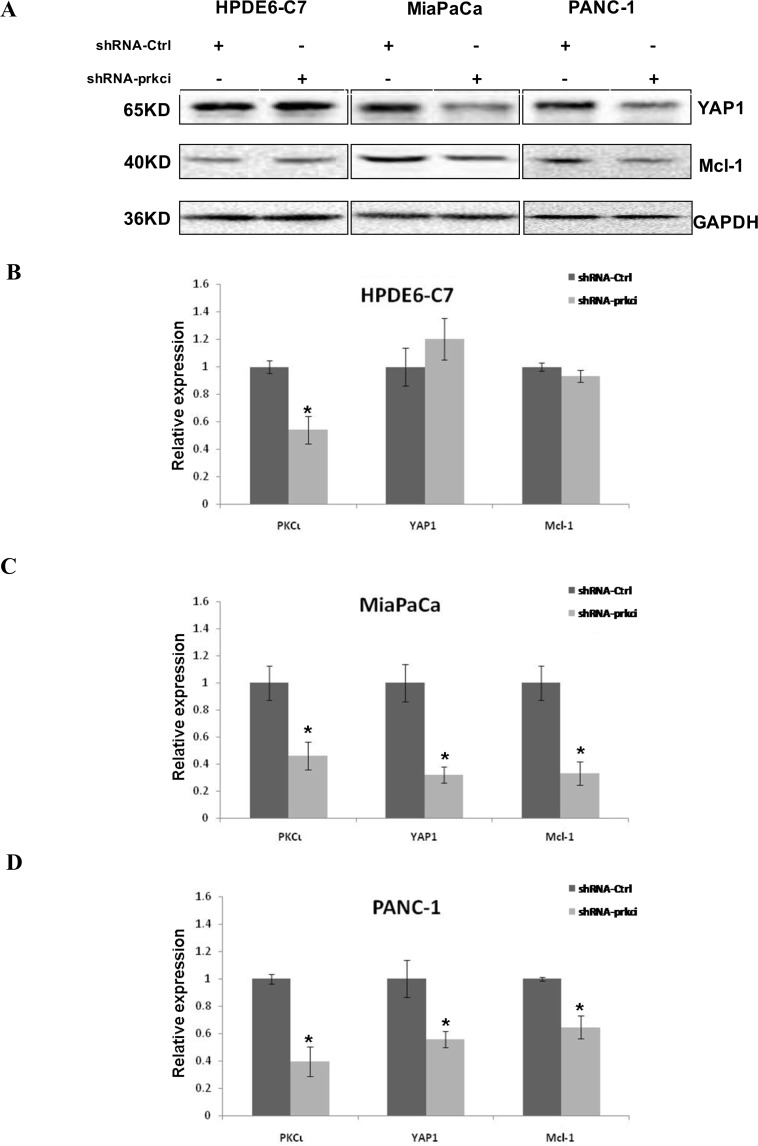
Downregulation of YAP1 and Mcl-1 in MiaPaCa and PANC-1, but not HPDE6-C7 cells upon PKCι knockdown by shRNA (**A)** Immunoblot analysis of YAP1 and Mcl-1 expression in HPDE6-C7, MiaPaCa and PANC-1 infected with lentivirus expressing shRNA-Ctrl or shRNA-prkci. (**B**) qRT-PCR analysis of PKCι, YAP1 and Mcl-1 levels in HPDE6-C7, (**C**) MiaPaCa and (**D**) PANC-1 expressing lentiviral shRNA-Ctrl or shRNA-prkci (Error bars, ± SD. *n =* 3. ^*^p < 0.05).

In concert with our discoveries made in cells treated with pharmacologic inhibitor of PKCι, the expression of YAP1 and Mcl-1 diminished in MiaPaCa and PANC-1, but not HPDE6-C7 when PKCι was knocked down by shRNA-prkci (Figure 3A–3D), further substantiating the important roles YAP1 and Mcl-1 play in PKCι-mediated cell death of PDAC harboring mutant Kras.

### Enforced expression of KRAS^G12C^ activates YAP1 signaling via PKCι in HPDE6-C7

**Figure 4:**
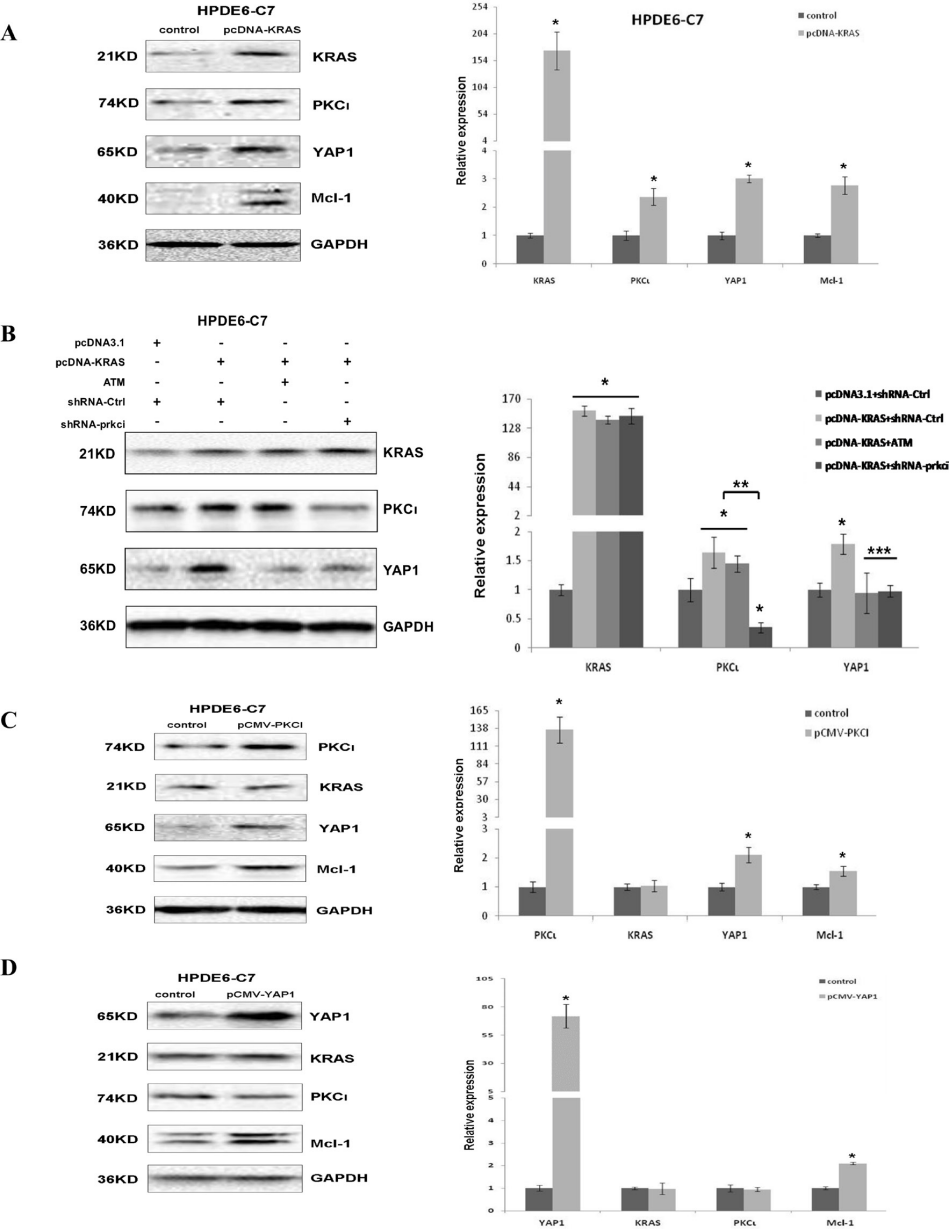
Ectopic expression of KRAS^G12C^ causes upregulation of YAP1 through PKCι in HPDE6-C7 (**A**) Immunoblot (left panels) and qRT-PCR (right panel) analyses of Kras, PKCι, YAP1 and Mcl-1 expression in HPDE6-C7 transiently transfected with void pcDNA3.1 vector (control) or pcDNA-KRAS. Data represent average of 3 replicate samples ± SD. ^*^p < 0.05. (**B**) Immunoblot detection (left panels) and qRT-PCR validation (right panel) of Kras, PKCι and YAP1 expression levels in HPDE6-C7 transiently transfected with control plasmids (void pcDNA3.1 and plasmid expressing shRNA-Ctrl), plasmids expressing KRAS^G12C^ (pcDNA-KRAS) and shRNA-Ctrl, or pcDNA-KRAS along with PKCι suppression by 0.1 mM ATM or shRNA-prkci. Data are mean of 3 replicates ± SD. ^*^p < 0.05 compared with control samples. ^**^p < 0.05 compared with Kras overexpression samples without shRNA-prkci interference. ^***^p < 0.05 compared with Kras overexpression samples without PKCι suppression. (**C**) Immunoblot (left panels) and qRT-PCR (right panel) analyses of PKCι, Kras, YAP1 and Mcl-1 expression in HPDE6-C7 transiently transfected with void pCMV-HA vector (control) or pCMV- PKCΙ (Error bar, ± SD. *n =* 3. ^*^p < 0.05). (**D**) Immunoblot (left panels) and qRT-PCR (right panel) analyses of YAP1, Kras, PKCι and Mcl-1 expression in HPDE6-C7 transiently transfected with void pCMV-HA (control) or pCMV-YAP1 (Error bar, ± SD. *n =* 3. ^*^p < 0.05).

Given that >90% PDAC, including MiaPaCa and PANC-1 cells, carry oncogenic Kras, we next sought to assess the interaction between Kras and PKCι-YAP1 signaling in pancreatic cells. First we transiently introduced a KRAS^G12C^-expressing plasmid into HPDE6-C7 and found that enforced expression of KRAS^G12C^ concurrently elevated the expression of PKCι and YAP1, accompanied by upregulation of YAP1 target *Mcl-1* gene (Figure 4A). Then we asked whether exogenous KRAS^G12C^ affected the expression of PKCι and YAP1 independently. To resolve that, we knocked down PKCι in HPDE6-C7 expressing KRAS^G12C^ using chemical or genetic method. Both immunoblot and qRT-PCR analyses demonstrated that YAP1 upregulation caused by ectopic KRAS^G12C^ was annulled in HPDE6-C7 cells upon PKCι inhibition (Figure 4B), suggesting that the KRAS^G12C^-induced YAP1 overexpression is associated with PKCι in HPDE6-C7 cells.

To further determine whether there is a feedback loop between Kras and PKCι-YAP1 signaling, we transiently transfected HPDE6-C7 with the plasmid expressing PKCι or YAP1. Immunoblot and qRT-PCR results showed that PKCι overexpression upregulated YAP1 and its downstream Mcl-1, but not Kras (Figure 4C). Similarly, YAP1 overexpression elevated Mcl-1, but not Kras and PKCι expression (Figure 4D), implying the lack of influence on Kras expression by PKCι or YAP1 in HPDE6-C7 cells.

Taken together, our experimental data establish that hyperactive Kras initiates the PKCι-mediated YAP1 upregulation, which may contribute to the development and maintenance of PDAC through enhancing the transcription of pro-proliferative target genes including *Mcl-1*.

### The Kras-PKCι-YAP1 signaling is present and required for the survival of PDAC harboring aberrant Kras

**Figure 5:**
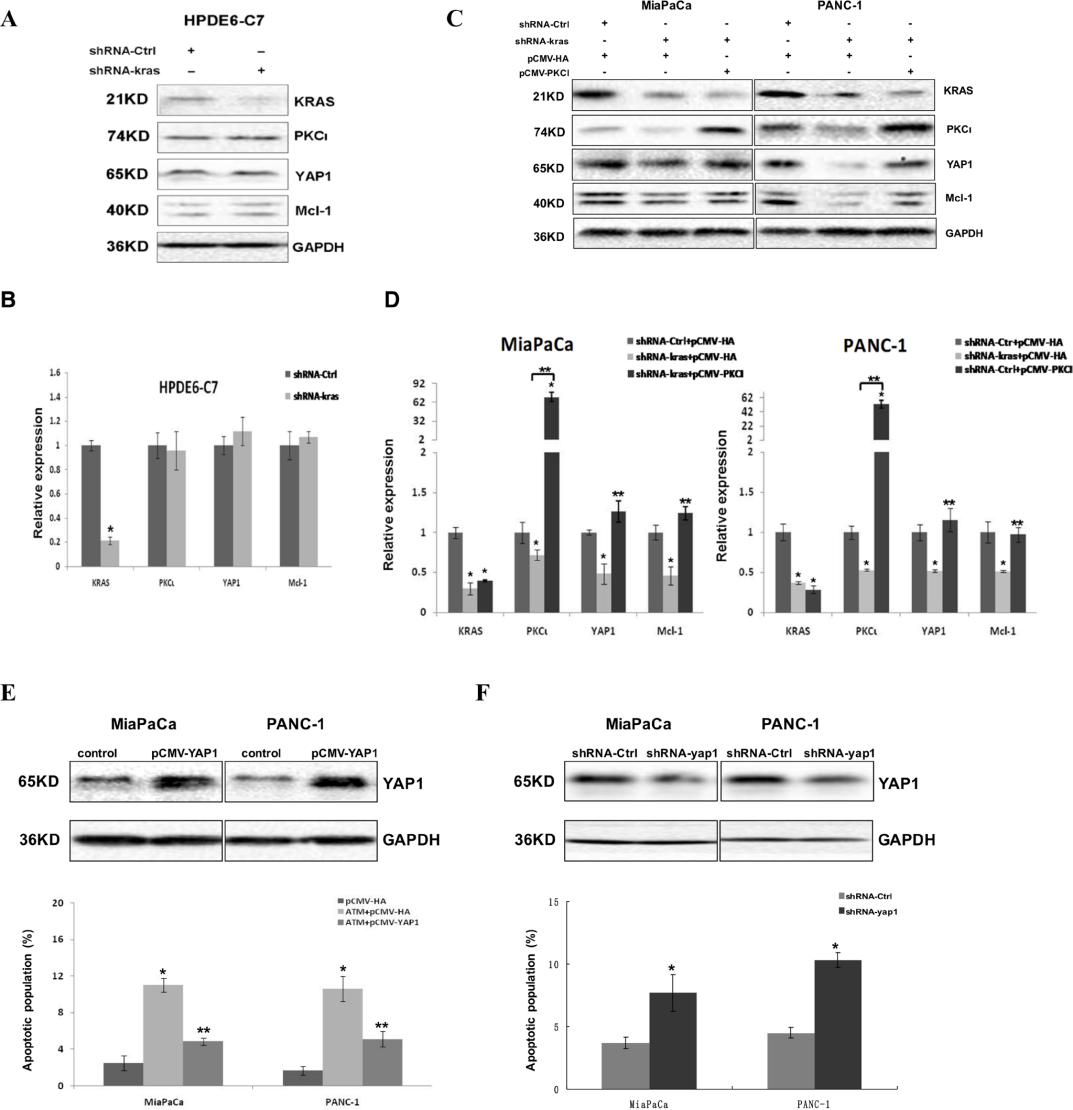
Kras-PKCι-YAP1 signaling pathway exists in Kras mutant pancreatic cancer cells and is crucial for the survival of these cells (**A**) Immunoblot and (**B**) qRT-PCR analyses of Kras, PKCι, YAP1 and Mcl-1 expression in HPDE6-C7 expressing control shRNA or shRNA-kras (Error bar, ± SD. *n =* 3. ^*^p < 0.05). (**C**) Immunoblot and (**D**) qRT-PCR analyses of Kras, PKCι, YAP1 and Mcl-1 expression in MiaPaCa and PANC-1 with Kras knockdown, or Kras knockdown in presence of PKCι overexpression. MiaPaCa and PANC-1 transfected with control plasmids (plasmid expressing control shRNA and void pCMV-HA) are used as controls (Error bar, ± SD. *n =* 3. ^*^p < 0.05 compared with control samples. ^**^p < 0.05 compared with Kras knockdown samples without PKCι overexpression). (**E**) Upper panels: Immunoblot detection of YAP1 overexpression in MiaPaCa and PANC-1 cells transfected with pCMV-YAP1 or void vector pCMV-HA (Control). Lower panel: Quantification of apoptotic cells in MiaPaCa and PANC-1 treated with control diluent, or with 0.1 mM ATM in or not in presence of YAP1 overexpression (Error bar, ± SD. *n =* 5. ^*^p < 0.05 compared with control cells. ^**^p < 0.05 compared with ATM-treated cells without YAP1 overexpression). (**F**) Upper panels: Immunoblot analysis of YAP1 expression in MiaPaCa and PANC-1 cells expressing lentiviral shRNA-Ctrl or shRNA-yap1. Lower panel: Quantification of apoptotic cells in MiaPaCa and PANC-1 infected with lentivirus producing shRNA-Ctrl or shRNA-yap1 (Error bar, ± SD. *n =* 5. ^*^p < 0.05).

To assess whether Kras-PKCι-YAP1 is a common signaling pathway in PDAC, we knocked down the expression of Kras with lentiviral shRNA in three pancreatic cell lines used in our study. The results revealed that Kras inhibition had little impact on the expression of PKCι, YAP1, or Mcl-1 in HPDE6-C7, whereas downregulating these genes in MiaPaCa and PANC-1 cells (Figure 5A–5D). Moreover, the reduced YAP1 expression was reversed by PKCι overexpression in Kras knockdown MiaPaCa and PANC-1 cells (Figure 5C and 5D), reinforcing that there resides a Kras-PKCι-YAP1 pathway in PDAC harboring aberrant Kras.

To further examine the role of YAP1 in apoptosis induced by PKCι knockdown in PDAC, we performed the gain/loss-of-function assays of YAP1 in pancreatic cancer cells. YAP1 overexpression was shown to partially rescue the MiaPaCa and PANC-1 from apoptosis induced by PKCι suppression, whereas YAP1 knockdown by shRNA phenocopied the PKCι suppression by eliciting apoptosis in MiaPaCa and PANC-1 cells (Figure 5E and 5F), providing evidence that PKCι knockdown induces apoptosis at least partially via YAP1, and Kras-PKCι-YAP1 signaling is essential for the survival of PDAC cells.

**Figure 6:**
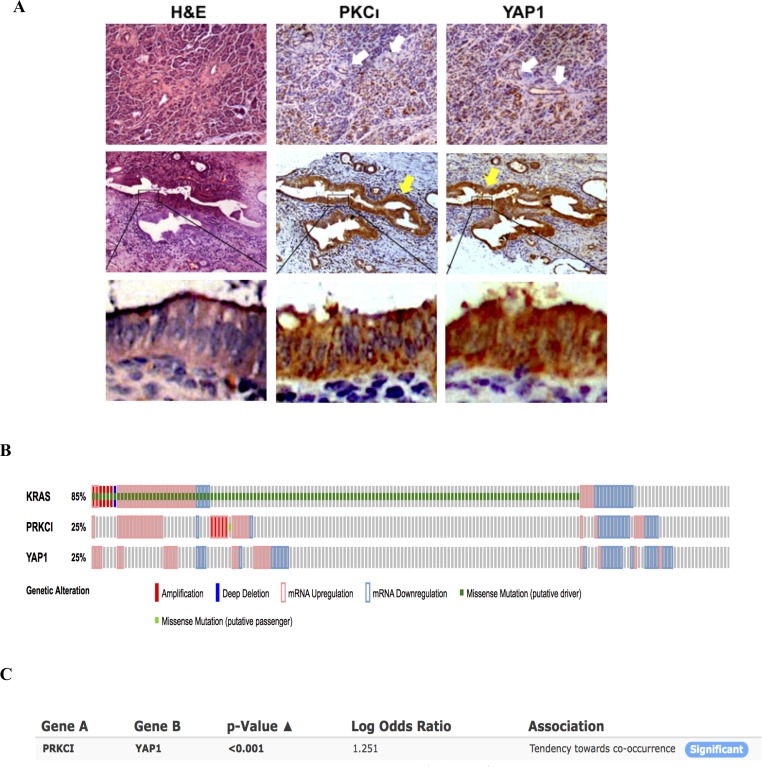
PKCι and YAP1 are concurrently upregulated in PDAC tissues (**A**) Representative immunohistochemical detection of upregulation of PKCι and YAP1 in the pancreatic lesions compared with the adjacent non-tumorous tissues. The anti-PKCι and anti-YAP1 staining of the pancreatic lesions are presented in series sections. The white arrows indicate negative to weak staining in normal ductal epithelium (the first panel). The yellow arrows indicate moderate to strong staining in the pancreatic lesions (the second panel). (**B**) Frequency of amplification (red bar), mutation (green bar), mRNA upregulation (pink frame) and mRNA downregulation (blue frame) for *Kras*, *PRKCI* and *YAP1* in pancreatic adenocarcinomas datasets (TCGA, Provisional). (**C**) Mutual exclusivity analysis of alterations in *PRKCI* and *YAP1* in pancreatic adenocarcinomas (TCGA, Provisional).

Next we evaluated the expression of PKCι and YAP1 in PDAC tissues. PKCι and YAP1 were stained immunohistochemically in serial sections of 15 PDAC samples collected in clinic. Of these 15 samples, 13 exhibit concurrent overexpression of PKCι and YAP1 compared to the matched adjacent non-tumorous tissues (Figure 6A). These results are in line with the previous findings showing that either PKCι or YAP1 is overexpressed in pancreatic cancer cells [17, 27], further portending the correlation between the overexpression of these two molecules in PDAC. Moreover, analysis of the Pancreatic Adenocarcinoma study in TCGA datasets reveals that among 178 samples with genomic sequencing and mRNA expression data, 151 (85%) have alterations in KRas (Figure 6B). Of these samples, only 15 show downregulated Kras mRNA expression without Kras mutation. The others (136 out of 151) are indicative of aberrantly high Kras activity by either containing constitutively active mu-Kras, or showing Kras mRNA upregulation, which is consistent with the Kras hyperactivity in the vast majority of pancreatic adenocarcinomas. Further Mutual Exclusivity analysis demonstrates a tendency towards co-occurrence between the *PRKCI* and *YAP1* mRNA alterations (*p* < 0.001) in pancreatic adenocarcinoma (Figure 6C), adding an extra line of evidence to the correlation between PKCι and YAP1 expression alterations, and the activation of PKCι-YAP1 signaling in Kras mutant PDAC.

### Stable transfection of HPDE6-C7 with KRAS^G12C^ activates PKCι-YAP1 signaling and sensitizes the cells to PKCι inhibition

**Figure 7:**
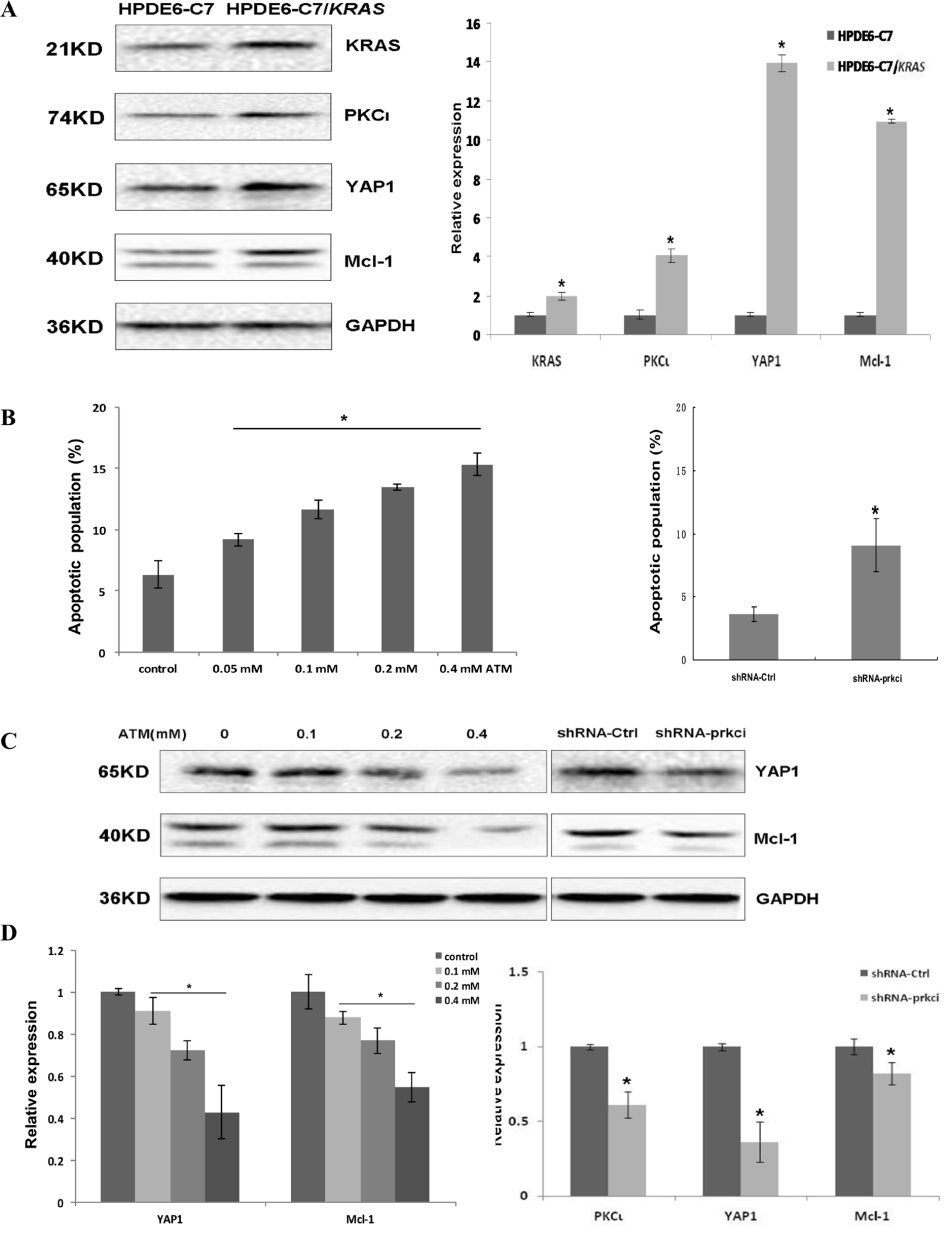
Stable transfection of HPDE6-C7 with KRAS^G12C^ renders the cells sensitivity towards PKCι inhibition (**A)** Immunoblot (left panels) and qRT-PCR (right panel) analyses demonstrating the upregulation of Kras, PKCι, YAP1 and Mcl-1 in HPDE6-C7/*KRAS* in comparison with its parental HPDE6-C7 cell line(Error bar, ± SD. *n =* 3. ^*^p < 0.05). (**B**) Quantification of apoptotic cells in HPDE6-C7/*KRAS* treated with increasing concentrations of ATM (left panel), and cells expressing lentiviral shRNA-Ctrl or shRNA-prkci (right panel. Error bar, ± SD. *n =* 5. ^*^p < 0.05). (**C**) Immunoblot and (**D**) qRT-PCR analyses of YAP1 and Mcl-1 expression in HPDE6-C7/*KRAS* treated with increasing concentrations of ATM, or infected with lentivirus expressing shRNA-Ctrl or shRNA-prkci (Error bar, ± SD. *n =* 3. ^*^p < 0.05).

To gain insight into whether activation of PKCι-YAP1 signaling by oncogenic Kras occurs in all PDAC with aberrant Kras, we engineered a HPDE6-C7/*KRAS* cell line by stably transfecting HPDE6-C7 with the plasmid expressing KRAS^G12C^. Immunoblot analysis revealed the overexpression of Kras, coinciding with upregulation of PKCι, YAP1 and Mcl-1 in HPDE6-C7/*KRAS* relative to parental HPDE6-C7 cells (Figure 7A). After being treated with ATM, HPDE6-C7/*KRAS* underwent apoptosis, along with reduced expression of YAP1 and Mcl-1 in a dose-dependent manner (Figure 7B–7D). Lentiviral shRNA-mediated PKCι knockdown also reduced YAP1 and Mcl-1 levels and caused apoptosis in HPDE6-C7/*KRAS* cells (Figure 7B–7D). These data support the notion that mu-Kras activates the PKCι-YAP1 signaling to support the development and survival of PDAC harboring oncogenic Kras.

## DISCUSSION

Most pancreatic ductal adenocarcinomas harbor Kras mutations. However, the comprehensive understanding of the oncogenic signaling pathways engaged by Kras, as well as their involvement in the initiation, maintenance and progression of PDAC is still not achieved, making developing targeted therapies for PDAC highly challenging. In the present study, we have attempted to approach this by searching for the PKC isozyme that has synthetic lethal effect with mutant Kras in PDAC. We identified that inhibition of PKCι, an atypical PKC isozyme, could induce apoptosis in PDAC cells carrying aberrant Kras, while exhibiting little impact on the survival of pancreatic epithelial cells containing normal Kras. PKCι is defined to be oncogenic and overexpressed in a wide spectrum of neoplasms including lung, prostate, pancreatic, colon, esophageal, liver, breast, ovarian and brain tumors [5, 24]. Importantly, PKCι is required for the Kras-driven transformation of colonic epithelial cell and bronchioalveolar stem cell, as well as the transformed growth and tumorigenesis of pancreatic cells [25–27], suggesting a relevance between PKCι’s oncogenic activity and the cellular context of mutant Kras. In our further exploration of the mechanisms underlying PKCι ablation-induced apoptosis in Kras mutant PDAC, we found that YAP1, a transcriptional co-activator and downstream effector of Hippo signaling pathway, was downregulated by PKCι inhibition in Kras mutant PDAC cells.

YAP1 is first discovered for its ability to associate with Yes and Src protein-tyrosine kinases, and demonstrated later to participate in a myriad of cellular events by activating the transcription of gene sets leading to cell proliferation, differentiation, development and apoptosis [28, 29]. The pivotal role of YAP1 in oncogenesis is highlighted by its association with the Hippo pathway, which is characterized as tumor suppressive in various cancers [28, 29]. Lats1/2 of Hippo pathway can phosphorylate and sequester YAP1 in cytoplasm for degradation, thereby abolishing its nuclear accumulation and shutting down its transcriptional activity. Recently, with accumulating evidence showing that YAP1 is responsive to multiple signaling pathways, YAP1 has emerged as an important nexus of signaling networks within the cells. The association of YAP1 with Kras or PKCι, and its implication in sustaining the survival of cancer cells have been documented in separate studies [22, 23, 30–33]. Zhang *et al.* pinpointed that YAP1 acting downstream of Kras is essential for the progression of PDAC [22]. More recently, Gruber *et al.* determined that hyperactive KRAS^G12D^ upregulated and activated YAP1 and TAZ (transcriptional co-activator with PDZ domain), which was required for the formation of acinar-to-ductal metaplasia (ADM) and pancreatic intraepithelial neoplasia (PanIN) driven by KRAS^G12D^ [30]. On the other hand, Wang *et al.* demonstrated that oncogenic PKCι unleashed YAP1 from its binding partner AMOT in cytoplasm, promoting the nuclear relocation and activation of YAP1 in ovarian cancer cells [23]. Nonetheless, the role of PKCι in Kras-mediated activation of YAP1 signaling in pancreatic cancer has not been investigated. In this study, we establish that oncogenic Kras upregulates and activates PKCι-mediated YAP1 signaling in PDAC. This finding advances our understanding of the biological effects of Kras, as well as the interactions among Kras, PKCι and YAP1 in PDAC.

Aside from activating downstream effectors, another output of oncogenic Ras signaling is to enhance the transcription of a wealth of molecules that are promotive to proliferation, including heparin-binding epidermal growth factor-like growth factor (HBEGF), transforming growth factor-α (TGF-α), amphiregulin (AREG), integrins and some growth factor receptors [34–36], while downregulating proteins that act oppositely [37, 38]. We discerned in this study that hyperactive Kras concurrently upregulated PKCι and YAP1 in pancreatic cells. The mechanism underneath the transcriptional regulation of PKCι by mutant Kras awaits further investigation. Nevertheless, we provide evidence that the upregulation of YAP1 by mutant Kras is mediated through PKCι, because the YAP1 overexpression induced by Kras was abrogated upon PKCι depletion, whereas PKCι overexpression elevated the expression of YAP1 in pancreatic cells. The regulation of downstream factors by oncogenic PKCι at transcriptional level has been reported before, with some of the key factors linking with Hedgehog (Hh) signaling pathways [39, 40]. PKCι was shown to phosphorylate and activate Gli1, a transcription factor involved in Hh signaling in basal cell carcinoma cells [39]. PKCι can also phosphorylate the transcriptional regulator of stemness, SOX2, enhancing its binding to the promoter of Hedgehog acyltransferase (HHAT) to facilitate the Hh target genes expression in lung squamous cell carcinoma [40]. Interestingly, hyperactive Hh signaling is defined as indispensable for the development of various tumors including PDAC [41–44], and YAP1 is identified as a major effector that can be upregulated and activated by Hh pathway in a subset of medulloblastomas, hepatic stellate cells and human and mouse pancreatic cancer cells [8, 45, 46]. In this study, our discovery that YAP1 is upregulated by Kras via PKCι raises the intriguing possibility that YAP1 may serve as a crucial signaling node that integrates the crosstalk among multiple, including Kras, Hippo and Hh signaling pathways during PDAC tumorigenesis.

The YAP1 signaling is featured by its reliance on the physiological context for cellular consequences, which are rendered primarily by its downstream target gene sets. Herein we verified that the level of Mcl-1, a pro-proliferative and anti-apoptotic protein belonging to Bcl-2 family [47] was consistently responsive to the change of YAP1 levels in the PDAC cell lines examined in our experiments, suggesting that *Mcl-1* is targeted by Kras-PKCι-YAP1 signaling in cells containing mu-Kras.

In summary, using PDAC cell lines and a isogenic cell model of human pancreatic cells, we demonstrate in the present study that mutant Kras upregulates PKCι, which stimulates the overexpression of YAP1 protein, leading to transactivation of pro-proliferative target gene *Mcl-1*, so to promote the tumorigenesis and maintenance of PDAC. Disruption of the Kras-PKCι-YAP1 signaling inhibits the growth and induces apoptosis in these cells. These results provide a rationale for further uncovering the molecules involved in this pathway and developing novel therapeutic strategies by targeting members of this signaling pathway in PDAC.

## MATERIALS AND METHODS

### Cells and reagents

Human pancreatic cancer cell lines MiaPaCa-2 and PANC-1 harboring aberrant *Kras* were purchased from the American Type Culture Collection (Manassas, VA, USA) and recently subjected to STR DNA profile analysis for authentication. The immortalized non-neoplastic pancreatic ductal epithelium cell line HPDE6-C7 was obtained from Gefan Biotech (Shanghai, China). The cells were maintained in Dulbecco’s Modified Eagle’s medium or RPMI-1640 medium (Yuanquan Biotech, Sichuan, China) supplemented with 10% fetal bovine serum (MHBio, Gansu, China) and cultured at 37° C in a humidified atmosphere containing 5% CO_2_. All the cells were routinely monitored and tested for mycoplasma contamination. The sodium aurothiomalate (ATM) was purchased from Sigma-Aldrich (St Louis, MO, USA). The antibodies used in this study include: anti-PKCι (BD Biosciences, San Jose, CA, USA), Kras, YAP1, Mcl-1, GAPDH (Zen Bioscience, Sichuan, China).

The small hairpin RNA (shRNA) sequences targeting human Kras, PKCι and YAP1 were reported before [27, 32] or obtained from Riobio Co. (Guangdong, China). The oligonucleotides containing small interference RNA sequences were ligated to pLentiLox 3.7 to produce lentiviral shRNAs. The cDNA of KRAS^G12C^ was cloned into pcDNA3.1/Hygro to generate plasmid pcDNA-KRAS, and the cDNAs of PKCι and YAP1 were inserted into pCMV-HA-N to generate expression plasmids pCMV-PKCI and pCMV-YAP1. Lipofectamine 2000 from Invitrogen (Rockville, MD, USA) was used for transfections.

### Cell growth assay

Typsin-dissociated cells were plated in 6-well plates. The number of the cells was counted in triplicate wells during the subsequent 6 to 8 days and plotted against time to generate cell growth curve.

The colorimetric MTT [3-(4,5-dimethylthiazol-2-yl)-2,5-diphenyltetrazolium bromide] metabolic activity assay was conducted according to the manufacturer’s instruction to determine the viability of the cells.

### Annexin V apoptosis detection assay

Cells were prepared and stained with Annexin V-PE apoptosis detection kit (Tianjin Sungene Biotech, Tianjin, China) as instructed by the manufacturer and subjected to flow cytometer analysis using Beckman coulter’s Cytomics FC 500.

### Quantitative real-time PCR (qRT-PCR)

The total RNAs were prepared using Trizol Reagent (Invitrogen, Rockville, MD, USA). The cDNAs were reversely transcribed and analyzed by CFX96™ Real-Time PCR Detection System (Bio-Rad, Hercules, CA, USA) to determine the expressions of target genes.

### Establishment of HPDE6-C7/*KRAS* cell line

HPDE6-C7 cells were transfected with pcDNA-KRAS and maintained in hygromycin-containing medium for 3 weeks to select for the HPDE6-C7/*KRAS* cell line that was stably transfected with pcDNA-KRAS.

### Immunoblot

Cell lysates were prepared and equal amounts of protein were resolved by sodium dodecyl sulfate-polyacrylamide (SDS) gel electrophoresis, transferred to nitrocellulose membranes, blocked with 5% non-fat milk, and then probed with primary and secondary antibodies before they were visualized with chemilluminescence.

### Patient samples and immunohistochemistry

Biospecimens were obtained from the West China hospital, Sichuan University under an approved institutional review board protocol. H&E-stained sections of the matched tumor and non-tumor tissues were analyzed by a pathologist to confirm the initial diagnosis and overall integrity of the tissue samples.

Tissues were fixed in 10% formalin overnight and embedded in paraffin. The tissues were deparaffinized by three changes of xylene and rehydrated in a graded ethanol series. The rehydrated tissue samples were rinsed in water and the antigens of the samples were retrieved in citrate buffer (pH 6.0). Slides were treated with 3% H_2_O_2_ for 5 min to reduce endogenous peroxidase activity, washed with PBS containing 0.5% (w/v) Tween 20, and incubated with PKCι and YAP1 antibodies. Images were captured and analyzed using Image-Pro Plus.

### Bioinformatic analysis

178 samples with genomic sequencing and mRNA expression data from the Pancreatic Adenocarcinoma study (TCGA, Provisional) in the cBio portal (www.cbioportal.org) were analyzed for mutation and mRNA expression alterations in *Kras*, *PRKCI* and *YAP1*. The *z*-score threshold for mRNA expression was set as 1.2.

### Statistic analysis

All data are presented as mean ± SD. One-way analysis of variance (ANOVA) was used to determine statistical significance of the results. *P* ≤ 0.05 was considered statistically significant.

## Abbreviations

AMOT: angiomotin
AREG: amphiregulin
ATM: aurothiomalate
EMT: epithelial-mesenchymal transition
HBEGF: heparin-binding epidermal growth factor-like growth factor
Hh: Hedgehog
HHAT: Hedgehog acyltransferase
Lats: large tumor suppressor
mu-Kras: mutated-K-ras
PKC: protein kinase C
PDAC: pancreatic ductal adenocarcinoma
TAZ: transcriptional co-activator with PDZ domain
TCGA: the Cancer Genome Atlas
TGF-α: transforming growth factor-α
YAP1: Yes-associated protein 1
